# Japanese multicenter prospective study investigating laparoscopic surgery for locally advanced rectal cancer with evaluation of CRM and TME quality (PRODUCT trial)

**DOI:** 10.1002/ags3.12592

**Published:** 2022-07-03

**Authors:** Ichiro Takemasa, Atsushi Hamabe, Masaaki Ito, Shuichiro Matoba, Jun Watanabe, Suguru Hasegawa, Masanori Kotake, Masafumi Inomata, Kazuki Ueda, Kay Uehara, Kazuhiro Sakamoto, Masataka Ikeda, Tsunekazu Hanai, Tsuyoshi Konishi, Shigeki Yamaguchi, Daisuke Nakano, Shigeru Yamagishi, Kenji Okita, Atsushi Ochiai, Yoshiharu Sakai, Masahiko Watanabe, Japan Society of Laparoscopic Colorectal Surgery

**Affiliations:** ^1^ Department of Surgery, Surgical Oncology and Science Sapporo Medical University Sapporo Japan; ^2^ Department of Colorectal Surgery National Cancer Center Hospital East Kashiwa Japan; ^3^ Department of Gastroenterological Surgery Toranomon Hospital Tokyo Japan; ^4^ Department of Surgery, Gastroenterological Center Yokohama City University Medical Center Yokohama Japan; ^5^ Department of Gastroenterological Surgery, Faculty of Medicine Fukuoka University Fukuoka Japan; ^6^ Department of Surgery Koseiren Takaoka Hospital Toyama Japan; ^7^ Department of Gastroenterological and Pediatric Surgery Oita University Faculty of Medicine Yufu Japan; ^8^ Department of Surgery Kindai University Faculty of Medicine Osakasayama Japan; ^9^ Division of Surgical Oncology, Department of Surgery Nagoya University Graduate School of Medicine Nagoya Japan; ^10^ Department of Coloproctological Surgery Juntendo University Faculty of Medicine Tokyo Japan; ^11^ Division of Lower Gastrointestinal Surgery, Department of Gastroenterological Surgery Hyogo College of Medicine Nishinomiya Japan; ^12^ Department of Surgery Fujita Health University, School of Medicine Toyoake Japan; ^13^ Department of Gastroenterological Surgery Cancer Institute Hospital of the Japanese Foundation for Cancer Research Tokyo Japan; ^14^ Department of Gastroenterological Surgery Saitama Medical University International Medical Center Hidaka Japan; ^15^ Department of Surgery Tokyo Metropolitan Cancer and Infectious Diseases Center Komagome Hospital Tokyo Japan; ^16^ Department of Surgery Fujisawa Municipal Hospital Fujisawa Japan; ^17^ Division of Biomarker Discovery, Exploratory Oncology Research & Clinical Trial Center National Cancer Center Kashiwa Japan; ^18^ Department of Surgery, Graduate School of Medicine Kyoto University Kyoto Japan; ^19^ Department of Surgery Kitasato University Kitasato Institute Hospital Tokyo Japan

**Keywords:** laparoscopy, magnetic resonance imaging, margins of excision, rectal neoplasms, total mesorectal excision

## Abstract

**Aim:**

In Japan, we have not been able to validate the results of laparoscopic surgery for locally advanced rectal cancer using the universal index “circumferential resection margin (CRM).” Previously, we established a semi‐opened circular specimen processing method and validated its feasibility. In the PRODUCT trial, we aimed to assess CRM in patients with locally advanced rectal cancer who underwent laparoscopic rectal resection.

**Methods:**

This was a multicenter, prospective, observational study. Eligible patients had histologically confirmed rectal adenocarcinoma located at or below 12 cm above the anal verge with clinical stage II or III and were scheduled for laparoscopic or robotic surgery. The primary endpoint was pathological CRM. CRM ≤1 mm was defined as positive.

**Results:**

A total of 303 patients operated on between August 2018 and January 2020 were included in the primary analysis. The number of patients with clinical stage II and III was 139 and 164, respectively. Upfront surgery was performed for 213 patients and neoadjuvant therapy for 90 patients. The median CRM was 4.0 mm (IQR, 2.1‐8.0 mm), and CRM was positive in 26 cases (8.6%). Univariate and multivariate analyses demonstrated that a predicted CRM from the mesorectal fascia of ≤1 mm on MRI was the significant factor for positive CRM (*P* = .0012 and *P* = .0045, respectively).

**Conclusion:**

This study showed the quality of laparoscopic rectal resection based on the CRM in Japan. Preoperative MRI is recommended for locally advanced rectal cancer to prevent CRM positivity.

## Graphical abstract

In this report, we validated circumferential resection margin (CRM) in patients with locally advanced rectal cancer who underwent laparoscopic rectal resection in a multicenter, prospective, observational study. This study showed the quality of Japanese laparoscopic rectal resection based on CRM for the first time, and MRI was demonstrated to be a crucial examination to predict positive CRM. These results would be quite significant in considering treatment strategy for locally advanced rectal cancer.

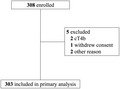

## 1 INTRODUCTION

Rectal resection still remains the mainstay in multidisciplinary treatment for locally advanced rectal cancer (LARC), and the quality of surgery is directly associated with postoperative local recurrence.^1^, ^2^, ^3^ Although the local recurrence rate for Dukes stage C was as high as 40% two decades ago, the establishment of total mesorectal excision (TME) since 1982 and adoption of neoadjuvant chemoradiotherapy as standard treatment have decreased the rate to approximately 5%.^4^, ^5^, ^6^ Different from Western countries, however, Japanese surgeons have adopted a unique strategy for LARC in which rectal resection with lateral lymph node dissection (LLND) is performed first regardless of preoperative staging, followed by postoperative chemotherapy.^1^ Despite this discrepancy, the long‐term results are comparable between Japan and Western countries.^7^

Although the treatment strategies evolved individually in Japan and in Western countries, the addition of LLND after neoadjuvant CRT can further prevent recurrence at the lateral pelvic cavity for patients with swollen nodes at baseline.^8^ This indicates that not all metastasized lateral lymph nodes can be eradicated with CRT alone, urging Western surgeons to indicate LLND for high‐risk patients. Japanese surgeons started to use neoadjuvant CRT to prevent local recurrence that cannot be prevented by LLND. The optimization of combination treatment with neoadjuvant CRT and LLND would be a key factor in completely preventing local recurrence after surgery for LARC.^9^

The indication for the combination of neoadjuvant CRT and/or LLND has to be discussed on the premise that the quality of rectal resection is appropriately ensured. However, the quality of Japanese surgery cannot have been evaluated in a pathological manner that is comparable to the method used in Western countries. According to the Japanese guidelines, the mesorectum is dissected off completely to harvest the perirectal lymph nodes and the rectum longitudinally opened to assess the macroscopic features of the tumor, which have long been emphasized for the evaluation of malignant potential,^10^, ^11^ leaving an inappropriate specimen for measuring the circumferential resection margin (CRM). Due to these differences, we have not been able to assess the pathological CRM to date, and the results of LARC treatment including LLND in Japan have not been accurately interpreted in Western countries. To resolve the inability to measure CRM in Japanese practice, we established a semi‐opened circular specimen processing method and validated its feasibility^12^, ^13^ to successfully measure pathological CRM. Based on this background, we aimed to prospectively assess the quality of laparoscopic surgery for LARC in Japan in a multicenter study by assessing the universal standard, CRM.

## 2 METHODS

### 2.1 Study design

This was a multicenter, prospective, observational study conducted in Japan. A total of 18 institutions from the Japan Society of Laparoscopic Colorectal Surgery participated in the study. Eligible patients were ≥20 years old, had histologically confirmed rectal adenocarcinoma located ≤12 cm above the anal verge with clinical stage II or III (T3N0M0, T1‐4aN1‐2M0), and scheduled for laparoscopic or robotic surgery. The deepest part of the tumor could be diagnosed as T4a, a tumor extending above the peritoneal reflection, and be regarded as eligible on the condition that the adjacent organ was not invaded. Rectal magnetic resonance imaging (MRI) was used for the assessment of T and N staging, and the clinical stage was assessed based on the images before neoadjuvant therapy. The indication for neoadjuvant therapy, surgical approaches, or LLND were at the discretion of each hospital. Exclusion criteria were as follows: a history of active double cancer (synchronous cancer, or metachronous cancer with disease‐free interval <5 years), cancer invading the adjacent organs, or psychiatric or addictive disorders that affected compliance with the protocol. The target sample size was 300, considering that previous randomized controlled trials comparing laparoscopic surgery to open surgery included approximately 200 patients for laparoscopic surgery,^14^, ^15^, ^16^ and that the estimated patients were 300 per year in participating institutions. All operations were controlled by credentialed surgeons in possession of an endoscopic surgical skill qualification system: qualified surgeon granted by the Japan Society for Endoscopic Surgery. The surgical approach could include not only conventional laparoscopic surgery (CLS) but also robotic‐assisted laparoscopic surgery (RALS). The study protocol was approved by the institutional review boards of the individual participating institutions. Written informed consent was obtained from all patients. This study was registered in the University Hospital Medical Information Network (UMIN) Clinical Trials Registry, Identification Number: UMIN00034364 (http://www.umin.ac.jp/ctr/index.htm).

### 2.2 Outcomes

The primary outcome was a pathological CRM measured using the semi‐opened circular specimen processing method. CRM was defined as negative if the distance between the closest tumor invasion and dissected plane was more than 1 mm. The secondary outcomes included the quality of TME, surgical and pathological findings, disease‐free survival, overall survival, and local recurrence rate. Our original plan was to report short‐term results, including CRM, which is the primary endpoint of this study, first and then demonstrate long‐term results after completing a follow‐up of all patients according to the previous randomized controlled trials, including ALaCaRT or Z6051.^5^, ^6^ The quality of the mesorectal excision was categorized as complete, nearly complete, or incomplete according to the Dutch TME trial: complete, smooth surface of mesorectal fascia with all fat contained in the enveloping fascia; nearly complete, the mesorectal envelope was intact except for defects no more than 5 mm deep, with no loss of mesorectal fat; incomplete, low bulk mesorectum with defects down onto the muscularis propria and/or a very irregular circumferential resection margin.^17^ The site of the lower border of the primary tumor (upper or lower) was categorized according to the subclassification of the 12 cm of rectum into equal halves. In assessing the clinical and pathological stage, the lateral lymph node was regarded as regional nodes according to the Japanese guidelines, and tumors with isolated metastasis to lateral lymph nodes were categorized as stage III.

### 2.3 Pathological assessment

The resected specimen was photographed from four directions to confirm the quality of the dissected mesorectal fascia: anteriorly, from right, from left, and posteriorly. The procedure for semi‐opened circular specimen processing was described in detail in our previous reports.^12^ Briefly, the area of the rectum between 2 cm above and below the borders of the rectal cancer is not incised and the corresponding mesorectum is left attached to the rectum in order to measure the CRM. In assessing the CRM, if the tumor invasion in lymph node metastasis, extramural vascular invasion (EMVI), or tumor deposit is closer to the dissected plane than the main tumor invasion, the closer distance is recorded as the CRM. The feasibility of this procedure for pathological assessment was previously verified in a multicenter, prospective, observational study, thereby confirming that the quality of semi‐opened circular specimen processing could be maintained in our study group.^13^

### 2.4 Statistical analysis

Statistical analyses were performed using JMP pro 15.1.0 software (SAS Institute, Cary, NC, USA). The results are expressed as the number of cases evaluated for categorical data, or as the median and interquartile range (IQR) for quantitative data. Univariate analyses were performed using Fisher's exact test or the Mann‐Whitney *U* test as appropriate. Factors associated with CRM positivity were assessed using a multivariate logistic regression model. p<0.05 was considered to be statistically significant.

## 3 RESULTS

### 3.1 Patient background and tumor characteristics

**FIGURE 1:**
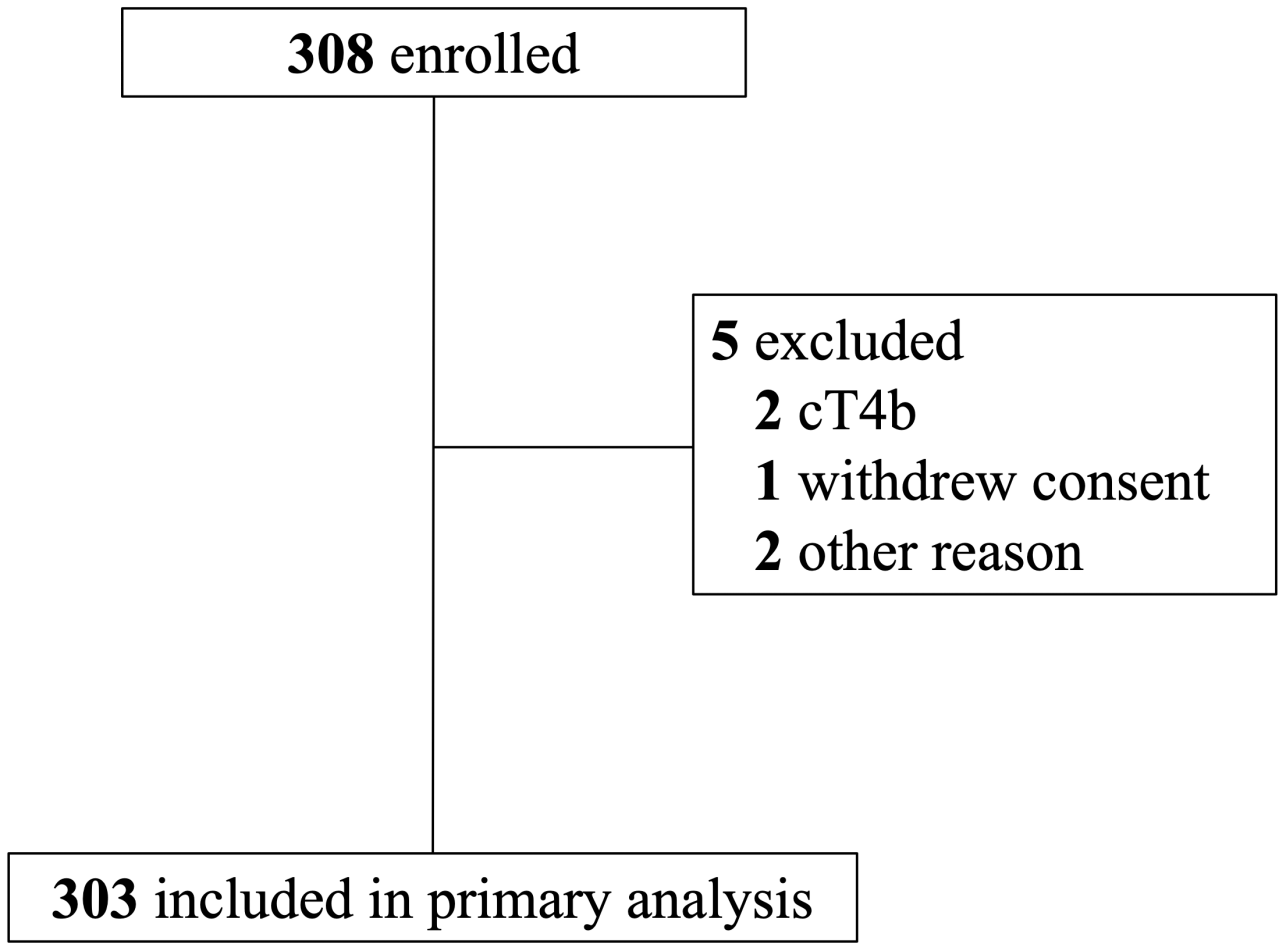
Flow of patient inclusion in the PRODUCT trial

Between August 2018 and January 2020, a total of 308 patients were enrolled in this study (Figure 1). Five patients were excluded after enrollment: two patients were ineligible because the tumor invaded the adjacent organs at baseline diagnosis, one patient withdrew consent, and two patients were ineligible for other reasons. Finally, 303 patients (202 males and 101 females) were included in the primary analysis.

Table 1 shows the patient and tumor characteristics. The median patient age was 66 years. At baseline, 139 cases were stage II and 164 stage III. Metastasis to lateral lymph nodes was suspected in 30 cases. The median distance from the lower border of the tumor to the anal verge was 70 mm (IQR, 40‐120 mm). Regarding neoadjuvant therapy, 213 patients received upfront surgery, and the other 90 patients underwent neoadjuvant therapy, including 27 cases of CRT, 45 cases of neoadjuvant chemotherapy (NAC), and 18 cases of total neoadjuvant therapy. No metastasis to distant organs was found after neoadjuvant therapy, and rectal resection could be performed for all cases undergoing neoadjuvant therapy.

**TABLE 1:** Patient and tumor characteristics

Patient characteristics (N = 303)	
Age, years	66 (56‐74)
Male sex	202 (66.7)
BMI, kg/m^2^	22.0 (20.2‐24.1)
ASA‐PS	
0‐1	288 (95.0)
2	15 (5.0)

Tumor characteristics at initial examination (N = 303)	
Distance from the anal verge, mm	70 (45‐100)
Location of the center of the tumor	
Anterior	85 (28.1)
Posterior	74 (24.4)
Right	31 (10.2)
Left	64 (21.1)
Circular	49 (16.1)
Tumor stage at baseline	
cT1	1 (0.3)
cT2	11 (3.6)
cT3	262 (86.5)
cT4a	29 (9.6)
Nodal status at baseline	
cN0	139 (45.9)
cN1	129 (42.6)
cN2	35 (11.6)
Suspected metastasis to lateral lymph node	30 (9.9)
Clinical stage at baseline	
Stage II	139 (45.9)
Stage III	164 (54.1)
Neoadjuvant therapy	
None	213 (70.3)
(C)RT	27 (8.9)
NAC	45 (14.9)
TNT	18 (5.9)

Tumor characteristics after neoadjuvant therapy (N = 90)	
Tumor stage after neoadjuvant therapy	
ycT0	1 (1.1)
ycT1	3 (3.3)
ycT2	18 (20.0)
ycT3	60 (66.7)
ycT4a	5 (5.6)
Not assessed	3 (3.3)
Nodal status after neoadjuvant therapy	
ycN0	58 (64.4)
ycN1	23 (25.6)
ycN2	6 (6.7)
Not assessed	3 (3.3)
Suspected metastasis to lateral lymph node	17 (18.9)
cCR	1 (1.1)

*Note*: Values are given as median (IQR) or n (%).

Abbreviations: ASA‐PS, American Society of Anesthesiologists physical status; BMI, body mass index; CR, complete response; CRT, chemoradiotherapy; IQR, interquartile range; NAC, neoadjuvant chemotherapy; RT, radiotherapy; TNT, total neoadjuvant therapy.

### 3.2 Surgical outcomes

Table 2 summarizes the results of the surgeries. CLS was performed for 167 cases and RALS for 136 cases. Regarding operative procedures, anterior resection was carried out for 240 cases, intersphincteric resection for 40 cases, abdominoperineal excision for 19 cases, and the Hartmann's operation for four cases. The transanal approach was utilized in 78 cases, and the distance from the anal verge was significantly shorter in these patients than those in whom the transanal approach was not used (median [IQR], 60 [40‐70] mm vs 70 [50‐100] mm, *P* < .0001). A diverting stoma was created for 177 cases.

**TABLE 2:** Operative results

Approach	
CLS	167 (55.1)
RALS	136 (44.9)
TaTME performed	78 (25.7)
Procedure	
LAR	240 (79.2)
ISR	40 (13.2)
APR	19 (6.3)
Hartmann	4 (1.3)
LLND	
None	213 (70.3)
Bilateral	76 (25.1)
Unilateral	14 (4.6)
Diverting stoma created	177 (62.1)
Operative duration, min	336 (256‐470)
Operative duration excluding LLND cases, min	320 (256‐393)
Blood loss, mL	12 (5‐50)
Blood loss excluding LLND cases, mL	7 (1‐50)
Open conversion	1 (0.3)
30‐day mortality	0 (0.0)
Length of hospital stay, days	14 (12‐21)
Grade 3‐4 postoperative complications	
Leakage	22 (7.3)
Bowel obstruction	8 (2.6)
Ileus	10 (3.3)
SSI (excluding leak)	10 (3.3)
Urological complication	10 (3.3)
Cardiovascular complication	4 (1.3)
Respiratory complication	3 (1.0)
Hemorrhage	3 (1.0)
Others	10 (3.3)

*Note*: Values are given as median (IQR) or n (%).

Abbreviations: APR, abdominoperineal resection; CLS, conventional laparoscopic surgery; IQR, interquartile range; ISR, intersphincteric resection; LAR, low anterior resection; LLND, lateral lymph node dissection; RALS, robotic‐assisted laparoscopic surgery; SSI, surgical site infection; TaTME, transanal total mesorectal excision.

The median operative duration was 336 minutes and blood loss 12 mL, whereas the operative duration was 320 minutes and blood loss 7 mL if excluding the cases in which LLND was performed. Regarding postoperative complications (Clavien‐Dindo ≥III), anastomotic leakage was found in 22 cases (rate of leakage, 7.3%), surgical site infection other than leakage in 10 cases, bowel obstruction in eight cases, paralytic ileus in 10 cases, and other complications in 30 cases. The median hospital stay was 14 days (12‐21 days).

### 3.3 Pathological outcomes

Pathological outcomes are shown in Table 3. The median length of pathologically assessed CRM, the primary endpoint of this study, was 4.0 mm (IQR, 2.1‐8.0 mm), and CRM was ≤1 mm in 26 cases, corresponding to a positivity rate of 8.6%. In these 26 cases, the CRM was positive at the site of the main tumor in 18 cases and positive at metastasized lymph nodes in six cases, a tumor nodule in one case, and at the intra‐lymphatic duct invasion in one case. Of the eight cases in which CRM was positive at a site other than the main tumor, there were no cases in which CRM status could be diagnosed correctly based on MRI. Regarding pathological staging, pCR was confirmed in 14 cases, and two, 76, 104, and 107 cases were stage 0, I, II, and III, respectively. The pCR rate corresponded to 15.6%. Metastasis to lateral lymph nodes was confirmed in 12 cases, including three cases with isolated metastasis to lateral lymph nodes. In evaluation of the TME quality, 293 cases were complete and 10 nearly complete. The distal resection margin (DRM) was positive in one case.

**TABLE 3:** Pathological results

Outcomes	All cases (N = 303)	Neoadjuvant therapy	
		No (N = 213)	Yes (N = 90)
CRM, mm	4.0 (2.1‐8.0)	4.0 (2.2‐8.0)	4.2 (2.0‐7.3)
With negative margin (>1 mm)	277 (91.4)	197 (92.5)	80 (88.9)
With positive margin (≤1 mm)	26 (8.6)	16 (7.5)	10 (11.1)
Tumoral region with CRM‐positivity (N = 26)			
Main tumor	18 (69.2)	9 (56.3)	9 (90.0)
Lymph node	6 (23.1)	5 (31.3)	1 (10.0)
Tumor nodule without residual lymph node structure	1 (3.8)	1 (6.3)	0 (0)
Intra‐lymphatic duct invasion	1 (3.8)	1 (6.3)	0 (0)
Total mesorectal excision			
Complete	293 (96.7)	205 (96.2)	88 (97.8)
Nearly complete	10 (3.3)	8 (3.8)	2 (2.2)
Incomplete	0 (0.0)	0 (0)	0 (0)
Tumor size, mm	36 (30‐45)	35 (30‐45)	38 (30‐45)
Pathological tumor stage			
pT0	16 (5.3)	0 (0)	16 (17.8)
pTis	2 (0.7)	0 (0)	2 (2.3)
pT1	10 (3.3)	6 (2.8)	4 (4.7)
pT2	84 (28.1)	59 (27.7)	25 (29.1)
pT3	176 (58.9)	139 (65.3)	37 (43.0)
pT4a	14 (4.7)	9 (4.2)	5 (5.8)
pT4b	1 (0.3)	0 (0)	1 (1.2)
Pathological nodal stage			
pN0	196 (64.7)	134 (62.9)	62 (72.2)
pN1	74 (24.4)	51 (23.9)	23 (22.2)
pN2	33 (10.9)	28 (13.1)	5 (5.6)
Metastasis to lateral lymph node	12 (4.0)	4 (1.9)	8 (8.9)
Pathological stage			
pCR	14 (4.6)	‐	14 (15.6)
Stage 0	2 (0.7)	0 (0)	2 (2.2)
Stage I	76 (25.1)	50 (23.5)	26 (28.9)
Stage II	104 (34.3)	84 (39.4)	20 (22.2)
Stage III	107 (35.3)	79 (37.1)	28 (31.1)
PRM, mm	120 (100‐150)	120 (100‐150)	130 (105‐163)
DRM, mm	20 (15‐30)	25 (15‐31)	20 (12‐30)

*Note*: Values are given as median (IQR) or n (%).

Abbreviations: CR, complete response; CRM, circumferential resection margin; DRM, distal resection margin; IQR, interquartile range; PRM, proximal resection margin.

### 3.4 Analysis of the risk factors for positive CRM

Table 4 summarizes the results of univariate analyses for the association between CRM positivity and the preoperative and operative variables. The predicted CRM from the mesorectal fascia being ≤1 mm on baseline MRI was found to be a significant risk factor for pathologically positive CRM (*P* = .0012). Among the 90 cases with neoadjuvant therapy, 84 underwent a second MRI to assess the therapeutic effect. The predicted CRM was positive on MRI in seven cases, four of which were diagnosed as pathologically positive. In contrast, among 77 cases with a negative predicted CRM on MRI, six were pathologically positive, which was significantly lower than the number predicted CRM to be positive (*P* = .0001). Although predicted CRM on MRI was suggested to be a risk factor after neoadjuvant therapy, we used the baseline MRI findings as candidate factors in the subsequent analysis because it was difficult to universally diagnose MRI after neoadjuvant therapy, which can be affected by therapeutic modifications.^18^ The multivariate analysis was carried out using the independent factors, including the predicted CRM from the mesorectal fascia on MRI and tumor stage cT4a, as well as tumor site and distance from the anal verge, which are the established risk factors for CRM positivity in the MERCURY II study.^19^ The cut‐off value for the distance from the anal verge was defined as 60 mm, considering the fact that low rectal cancer located ≤6 cm from the anal verge was regarded as a definite risk factor for CRM positivity in rectal cancer.^19^ As shown in Table 5, a predicted CRM from the mesorectal fascia ≤1 mm on baseline MRI was demonstrated to be a significant factor, but the other factors were not shown to be related to the CRM positivity.

**TABLE 4:** Univariate analyses of the risk factors for positive CRM

	CRM‐negative (N = 277)	CRM‐positive (N = 26)	*P*‐value
Patient characteristics			
Median age (IQR), years	66 (56‐74)	67 (53‐76)	.7895
Sex, n (%)			.1907
Male	188 (67.9)	14 (53.8)	
Female	89 (32.1)	12 (46.2)	
Median BMI (IQR), kg/m^2^	22.0 (20.4‐24.1)	20.7 (19.5‐23.7)	.1906
Tumor characteristics at initial examination			
Tumor stage, n (%)			.0876 (T1‐3 vs T4a)
cT1	1 (0.4)	0 (0.0)	
cT2	11 (4.0)	0 (0.0)	
cT3	241 (87.0)	21 (80.8)	
cT4a	24 (8.7)	5 (19.2)	
Nodal status, n (%)			.8375 (N0 vs N1‐2)
cN0	128 (46.2)	11 (42.3)	
cN1	117 (42.2)	12 (46.2)	
cN2	32 (11.6)	3 (11.5)	
Distance from the anal verge			.1536
>60 mm	149 (53.8)	10 (38.5)	
≤60 mm	128 (46.2)	16 (61.5)	
Neoadjuvant therapy		0.3691	
Yes	80 (28.9)	10 (38.5)	
No	197 (71.1)	16 (61.5)	
Predicted CRM from mesorectal fascia on MRI			.0012^*^
>1 mm	245 (92.1)	17 (68.0)	
≤1 mm	21 (7.9)	8 (32.0)	
Tumor site			
Anterior	121 (43.7)	13 (50.0)	.5421
Not anterior	156 (56.3)	13 (50.0)	
Surgery			
TaTME			
Yes	74 (26.7)	4 (15.4)	.2479
No	203 (73.3)	22 (84.6)	
Approach			
Laparoscopy	154 (55.6)	13 (50.0)	.6812
Robot	123 (44.4)	13 (50.0)	
Procedure		0.5995	
LAR	221 (79.8)	19 (73.1)	
ISR	36 (13.0)	4 (15.4)	
APR	16 (5.8)	3 (11.5)	
Hartmann	4 (1.4)	0 (0.0)	

*Note*: Values are given as median (IQR) or n (%). MRI was not performed in 13 cases.

Abbreviations: APR, abdominoperineal resection; BMI, body mass index; CRM, circumferential resection margin; IQR, interquartile range; ISR, intersphincteric resection; LAR, low anterior resection; TaTME, transanal total mesorectal excision. ^*^ indicates statistically significant.

**TABLE 5:** Multivariate analysis of the risk factors for positive CRM

	OR [95% CI]	*P*‐value
Tumor stage		
cT1‐T3	reference	.2024
cT4a	2.19 [0.656‐7.30]	
Predicted CRM on MRI		
>1 mm	reference	.0045^*^
≤ 1 mm	4.33 [1.58‐11.9]	
Distance from the anal verge		
>60 mm	reference	.3160
≤60 mm	1.59 [0.643‐3.93]	
Tumor site		
Not anterior	reference	.8381
Anterior	1.09 [0.459‐2.61]	

Abbreviations: CI, confidence interval; CRM, circumferential resection margin; OR, odds ratio.^*^ indicates statistically significant.

## 4 DISCUSSION

In this study, we assessed the pathological CRM for LARC operated on patients in Japan in a multicenter prospective study using the semi‐opened circular specimen processing method. Until now, we have not been able to validate the oncological results of rectal cancer treatment in Japan by directly comparing it to the results in Western countries due to the lack of CRM data, which is a definitive predictive marker of future relapse. This restriction has been a hindrance for a number of past Japanese clinical studies to be appraised internationally, which needed to be solved by defining the tumor characteristics using CRM. Therefore, this study is important, as using the semi‐opened circular specimen processing method to assess CRM could demonstrate the results of laparoscopic surgery for LARC in Japan, where a different strategy has been adopted compared to Western countries, and also show the rate of positive CRM in Japan, which can be used as a reference in future trials. This method is beneficial in that we can easily harvest the tumor sample from the resected specimen, which would be mandatory in the era of genome medicine.

There have been four randomized controlled trials comparing the results of laparoscopic surgeries for LARC to the results of open surgeries. Although the COREAN trial^15^ and COLOR II trial^20^ showed favorable results of laparoscopic surgery, the ALaCaRT trial and Z6051 trial could not demonstrate the non‐inferiority of laparoscopic surgery to open surgery by validating the successful resection rate based on several factors, including the assessment of CRM positivity, raising concerns about the application of laparoscopic surgery to LARC.^14^, ^16^ In analyses of the long‐term results of the above two trials, laparoscopic surgery was eventually demonstrated not to be a risk factor for disease‐free survival, though pathological CRM was the single, poor prognostic factor.^5^, ^6^ These data suggested that, in the era of multimodal therapy for locally advanced rectal cancers, obtaining a CRM of more than 1 mm is the most crucial factor to achieving sufficient curability regardless of the surgical approach. In this regard, assessment of CRM is essential to validate the surgical quality and predict patient prognosis. The CRM in this study was shown to be 8.6%, and approximately 70% of the cases were positive at the site of the main tumor. Intriguingly, one‐third of the patients were positive at a site other than the main tumor, suggesting the importance of preoperative assessment in tumor expansion in the mesorectum. The positivity rate of CRM was lower in the COREAN (2.1%) and ALaCaRT (6.7%) trials, and higher in the COLOR II (9.5%) and Z6051 (12.1%) trials, compared to the rate in the PRODUCT trial. In interpreting the differences in CRM positivity, the differences in the enrolled patients should be considered. In contrast to the other trials, neoadjuvant therapy was administered in as few as 30% of cases in the presented study, and NAC was used in roughly half of the patients. Furthermore, more advanced cases were enrolled in this study based on the pathological tumor stage, and more than 50% of the cases had T3‐T4. The definition of CRM positivity also differed. CRM is defined as positive if ≤2 mm in the COLOR II trial, <1 mm in the ALaCaRT trial, or ≤1 mm in the COREAN and Z6051 trial and this study. The positive CRM rate of 8.6% in this study should be considered by taking the above‐mentioned differences into account. There could be room to further improve the positive rate and some measures need to be taken, including the application of multimodal therapy for high‐risk patients. Based on the sufficient treatment for local control, the indication of the intensified treatment to prevent distant metastasis, such as total neoadjuvant therapy, should be discussed.

This study has shown that the tumor proximity to the mesorectal fascia assessed on baseline MRI is an independent risk factor for positive CRM. In the current Japanese guidelines, we cannot see any recommendation for implementing MRI as a preoperative work‐up in the treatment of rectal cancer, which is a mandatory exam in Western countries.^1^, ^2^, ^3^ In Japan, upfront surgery has been a mainstay for advanced rectal cancer regardless of the individual tumor status; thus, the importance of finding various malignant features to assess on MRI has long been undervalued. However, given that the tumor proximity to the mesorectal fascia on MRI has been demonstrated to be a definitive risk factor for positive CRM in the setting of tertiary referral hospitals in Japan, we argue that baseline MRI assessment should be carried out for LARC similar to in Western countries. The number of MRIs installed in Japan is quite high, though the protocol or procedure for acquiring high‐resolution images for LARC is not standardized.^21^ Thus, the surgical plane to be dissected or the indication for neoadjuvant therapy should be evaluated preoperatively based on MRI findings. In the ESMO guidelines, EMVI, a well‐known risk factor for distant metastasis or positive CRM, has to be assessed preoperatively to decide on the multimodal therapy. However, we did not evaluate EMVI in this study because the assessment for EMVI is not currently general in Japan.^2^, ^18^, ^22^ We consider that mrEMVI should be validated in a future sub‐analysis using these datasets after achieving consensus on diagnosis using MRI with expert radiologists, and that the basis for interpreting EMVI on MRI should be constructed. Moreover, considering the fact that CRM was positive at a site other than the main tumor in one‐third of cases, it would be ideal if the accuracy of MRI for diagnosing the tumor extension beyond the main tumor is improved even though it is a challenging task.

Various types of surgery were utilized in the analyzed cases. Today, we can adopt several approaches other than CLS in the field of minimally invasive surgery, including the advancement and spread of RALS and transanal TME. Both approaches aim to improve the quality of TME and secure the achievement of negative CRM.^23^, ^24^, ^25^, ^26^ This study showed equivalent results for several approaches, including CLS. The ROLARR trial could not show the superiority of RALS compared to CLS in regards to the rate of open conversion as a primary endpoint, as well as CRM.^27^ However, in the ROLARR trial, the strict regulation was not set up for an operator of RALS, which was considered a possible factor affecting the results. In the PRODUCT study, not a few RALS were performed by surgeons on the learning curve. To validate the efficiency of RALS for LARC, we are now conducting a multicenter prospective observational study to analyze CRM as a primary endpoint (VITRUVIANO trial, UMIN000039685) in which the surgeons have to be credentialed based on the number of performed cases not being less than 40. With regard to transanal TME, this procedure is considered beneficial in securely obtaining a free DRM, as well as CRM for the tumor close to the anal verge.^25^, ^28^ Despite significance not being found, the distance from the anal verge was shorter in cases with a transanal approach, suggesting that it may offer a chance to prevent CRM positivity in such cases.

The present study has several limitations. First, the single‐arm cases cannot be compared to the results of open surgery. Second, the long‐term results were not analyzed in this study; therefore, we cannot conclude that the Japanese laparoscopic surgeries are feasible for LARC. Validation of the long‐term results of the included patients is going to be carried out in a future ancillary analysis.

In conclusion, this study has demonstrated the results of Japanese CRM after laparoscopic rectal resection for LARC for the first time. CRM positivity was found in 8.6% of cases, one‐third of which were positive at a site other than the main tumor. MRI should be carried out for patients with possible LARC at baseline to decide on the surgical plane. The rate of positive CRM shown in this study will play an important role as a reference value in future studies.

## DISCLOSURE

Funding: This study received no funding.

Conflicts of Interest: Tsuyoshi Konishi reported lecture fees from Johnson & Johnson Japan and Medtronic Japan. The other authors declare no conflicts of interest. Ichiro Takemasa, Jun Watanabe, Masafumi Inomata, and Kay Uehara are editorial members of *Annals of Gastroenterologial Surgery*.

Author contributions: Conceptualization: Ichiro Takemasa, Yoshiharu Sakai, Masahiko Watanabe; Methodology, patient enrollment, formal analysis and investigation: Ichiro Takemasa, Atsushi Hamabe, Masaaki Ito, Shuichiro Matoba, Jun Watanabe, Suguru Hasegawa, Masanori Kotake, Masafumi Inomata, Kazuki Ueda, Kay Uehara, Kazuhiro Sakamoto, Masataka Ikeda, Tsunekazu Hanai, Tsuyoshi Konishi, Shigeki Yamaguchi, Daisuke Nakano, Shigeru Yamagishi, Kenji Okita, Atsushi Ochiai, Yoshiharu Sakai, Masahiko Watanabe; Principal investigators in participating hospitals of this multicenter study: Ichiro Takemasa, Masaaki Ito, Shuichiro Matoba, Jun Watanabe, Suguru Hasegawa, Masanori Kotake, Masafumi Inomata, Kazuki Ueda, Kay Uehara, Kazuhiro Sakamoto, Masataka Ikeda, Tsunekazu Hanai, Tsuyoshi Konishi, Shigeki Yamaguchi, Daisuke Nakano, Shigeru Yamagishi, Yoshiharu Sakai, Masahiko Watanabe; Writing ‐ original draft preparation: Atsushi Hamabe, Kenji Okita; Writing ‐ review and editing: Ichiro Takemasa, Yoshiharu Sakai, Masahiko Watanabe.

Compliance and Ethical Standards: The protocol for this study was approved by the institutional review boards of the participating hospitals. Written informed consent was obtained from all patients. Japanese multicenter prospective study investigating laparoscopic surgery for locally advanced rectal cancer with evaluation of CRM and TME quality (PRODUCT trial) was registered in the University Hospital Medical Information Network (UMIN) Clinical Trials Registry, Identification Number: UMIN00034364 (http://www.umin.ac.jp/ctr/index.htm).
